# Knowledge, attitude, and practice towards food safety amongst school children, food handlers and consumers: protocol for a pre-post longitudinal study in North East India

**DOI:** 10.3389/fpubh.2025.1643443

**Published:** 2025-10-17

**Authors:** Rashmi Savant, Rajkumar James Singh, Suranjana Chaliha Hazarika, Tapan Majumdar, Karma G. Dolma, Sarangthem Indira Devi, Tapan Kumar Dutta, Valerie Lyngdoh, Dilem Modi, Swagnik Roy, Rajkumari Mandakini Devi, Hosterson Kylla, Megongusie Meru, Samaresh Das, Shalony Roy, Thandavarayan Ramamurthy, Priyanshu Das, Madhuchhanda Das

**Affiliations:** ^1^Indian Council of Medical Research, New Delhi, India; ^2^Gauhati Medical College and Hospital, Guwahati, Assam, India; ^3^Agartala Government Medical College, Agartala, Tripura, India; ^4^Sikkim Manipal Institute of Medical Sciences, Gangtok, India; ^5^Institute of Bioresources and Sustainable Development (IBSD), Imphal, India; ^6^College of Veterinary Sciences & Animal Husbandry, Central Agricultural University, Aizawl, Mizoram, India; ^7^North Eastern Indira Gandhi Regional Institute of Health and Medical Sciences, Shillong, Meghalaya, India; ^8^Bakin Pertin General Hospital & Research Institute, Pasighat, Arunachal Pradesh, India; ^9^Zoram Medical College, Aizawl, Mizoram, India; ^10^College of Veterinary Sciences & Animal Husbandry, Central Agricultural University(I), Jalukie, Nagaland, India; ^11^State Disease Diagnostic Laboratory, Department of Animal Husbandry and Veterinary, Government of Meghalaya, Shillong, India; ^12^Christian Institute of Health Sciences and Research, Dimapur, Nagaland, India; ^13^Centre for Development of Advanced Computing (C-DAC), Kolkata, India; ^14^National Institute for Research in Bacterial Infections (NIRBI), Indian Council of Medical Research, Kolkata, India; ^15^Kasturba Medical College, Manipal, Karnataka, India

**Keywords:** knowledge, attitudes, practices, foodborne infections, food safety, awareness, public health

## Abstract

**Background:**

The North Eastern states of India exhibit a rich diversity of cultural, geographical, and traditional food practices, which, while unique and valuable, contribute to an increased susceptibility to foodborne and waterborne diseases. While these practices contribute to the region’s identity, inadequate food safety measures increase the risk of foodborne diseases, constituting a pressing public health issue.

**Objective:**

This study aims to assess the knowledge, attitudes, and practices (KAP) related to food safety among school children, food handlers, and consumers in eight North Eastern (NE) states of India. It also evaluates the changes in KAP scores across all three study groups following an educational intervention program.

**Methods:**

A pre-post quasi experimental longitudinal study design involving 11 NE centres under the ICMR FoodNet program and comprising three waves:

- *Wave 1*: Baseline cross-sectional KAP assessment among school-going children (studying in 9^th^ and 10^th^ standard), food handlers, and consumers by using structured questionnaires adapted from WHO and CDC guidelines.
- *Wave 2*: Implementation of an educational intervention in all three study groups only, guided by a checklist. No intervention for control groups.
- *Wave 3*: Post-intervention quasi-experimental assessment of both the study and control groups to evaluate changes in KAP at one-month post-intervention, and 6 months post-intervention.

**Intended use of results:**

The study is expected to enhance knowledge and promote sustainable hygiene practices among participants, reduce high-risk behaviors, and foster community-level dissemination of food safety knowledge. Findings will contribute to evidence-based policymaking and support the development of a Social and Behavior Change Communication (SBCC) model for food safety. Insights from this pilot study will provide an adaptable framework to generate food safety awareness data in other regions with diverse cultural and geographical contexts. The mobile app developed for this KAP study will act as a ‘scalable tool’ by enabling field workers to efficiently collect and submit standardized KAP data across diverse settings, thereby improving consistency and moderating the logistical burden often associated with such large-scale field surveys. If effective, the app can support data collection nationwide, extending its utility beyond North East India.

## Graphical abstract

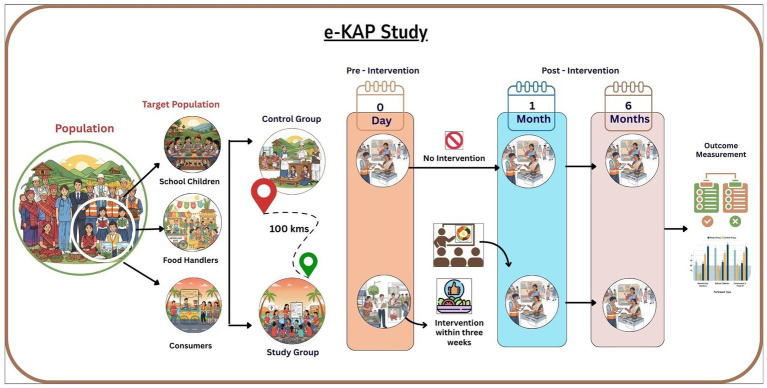

## Introduction

Food borne diseases cause a significant public health problem and are responsible for the loss of 33 million years of healthy life annually, worldwide (1). Consumption of contaminated food causes more than 200 different types of diseases. As per the World Health Organisation (WHO) report, foodborne diseases (FBDs) affect one in ten people globally, and poor food safety and hygiene practices cause 600 million cases and 420,000 deaths each year (2). Waterborne diarrhoeal diseases are known to cause nearly 829,000 deaths across the world, every year (3). The complex nature of food sources, cultural diversity of food preparation and handling, as well as the possibility of transmission of pathogens from production level to consumption level are some of the main causes for the occurrence of foodborne diseases (2). Likewise, unsafe drinking water and unhygienic sanitation practices are primarily associated with waterborne diseases. Rotavirus is responsible for over half a million deaths in a year throughout the world (4). According to the WHO report, every year nearly 1.3–4.0 million cases of cholera are detected and result in nearly 21,000–1,43,000 deaths on a global scale (5). Norovirus, the key causative organism for acute and sporadic gastroenteritis causes 210,000 fatalities and 685 million cases of illness, annually (6). A global burden study on typhoid and paratyphoid reported an estimated 7,154,555 new cases and 93,333 deaths in the year 2021 (7).

A study involving 5,246 adults in the USA reported that the prevalence of chronic diarrhoea was 6.6% (8). The prevalence and incidence of diarrhoea in India are notably higher than those reported in high-income countries like the United States and countries in Western Europe. A cross-sectional study in the semi-urban and rural regions of South Canara district reported a period prevalence of diarrhoea of 12% among adults (9). Similarly, a study conducted in Delhi involving 6,285 individuals living in temporary shelters found an overall diarrhoea incidence of 29.1 cases per 1,000 persons (10). The comparatively higher rates observed in Indian settings can be attributed to factors such as inadequate sanitation, dearth of clean water, and challenges related to food safety.

In India after the introduction of ‘Swachh Bharat Mission’ (SBM) in 2014 and ‘Open Defection Free India Mission’, burden of diarrhoeal diseases and outbreaks reduced significantly (11). A study on infant mortality across 640 districts in India over a ten-year period found that the large-scale sanitation program contributed to averting at least 60,000 to 70,000 deaths each year among children under 5 years of age (12).

To strengthen the effect of national programs for further reduction of food and water borne diseases, active involvement of public is much needed. It is well documented that awareness and practices are a strong tool to improve public health dramatically (13). Programs like the Integrated Disease Surveillance Programme (IDSP) and Food Safety and Standards Authority of India (FSSAI) “Eat Right India” campaign also emphasize education and awareness, promoting safe food practices.

In view of the lifestyle and food habits of North-East people, the Indian Council of Medical Research (ICMR) launched its first foodborne disease surveillance network, ICMR FoodNet, under the mission project titled “Surveillance of Foodborne Pathogens (FBP) from Northeast India (Phase I & II).” This initiative focuses on the eight North-Eastern states of India, establishing a robust and systematic surveillance system and research network along with an awareness program across 11 North-East centres.

Preliminary findings from the ICMR FoodNet project indicate significant gaps in KAP regarding food safety among the region’s diverse communities. Thus, the current KAP study is focused on food safety awareness and practice to understand knowledge gaps, perception, and food practice behavior in different groups of people. To the best of our knowledge, this awareness program will be the first of its kind to cover three most critical target groups of the population, i.e., food handlers (homemakers and street food vendors), school children, and consumers. Food handlers play a crucial role in the spread of food borne infections, whereas, school children are the future citizen and are more prone to be infected by food borne pathogens. Additionally, consumers need to be aware of foodborne infections and safe hygiene practices so that they can refrain from purchasing unhealthy/unhygienic food items.

Findings from this study can be used to generate an effective awareness model that can be implemented through a national program to reduce food and water-borne diseases and outbreaks in the country.

### Aims and objectives

To assess KAP on food safety and hygiene in the North-east region of India amongst School children, food handlers and consumers (Wave 1). – To implement an educational intervention in all three groups, guided by a checklist (Wave 2). – To perform a post-intervention quasi-experimental assessment and evaluate the changes in KAP of all three groups (Wave 3).

## Methods

### Study design

**Figure 1:**
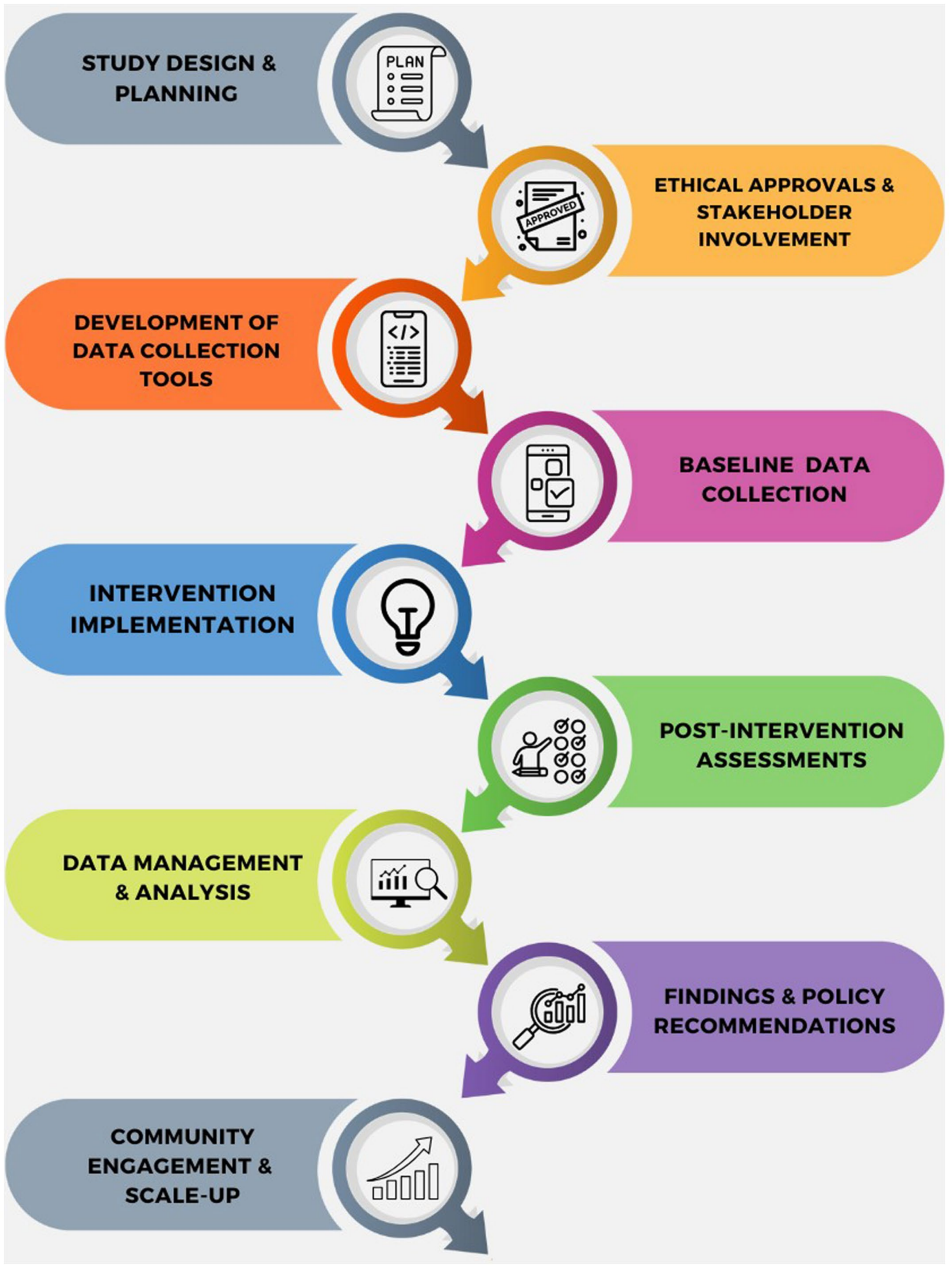
KAP awareness initiative—work flow.

A pre-post quasi experiment study following a quantitative approach through a KAP survey focused on food safety and hygiene, utilising a structured and standardised questionnaire to capture data (Figure 1) was designed.

Data will be collected at three-time points: one pre-intervention (a cross-sectional KAP for baseline data collection) and two post-interventions. Participants from both urban and rural locations will be included wherever possible, maintaining integrity and diversity across sectors and locations within each study area.

### Study area

**Figure 2:**
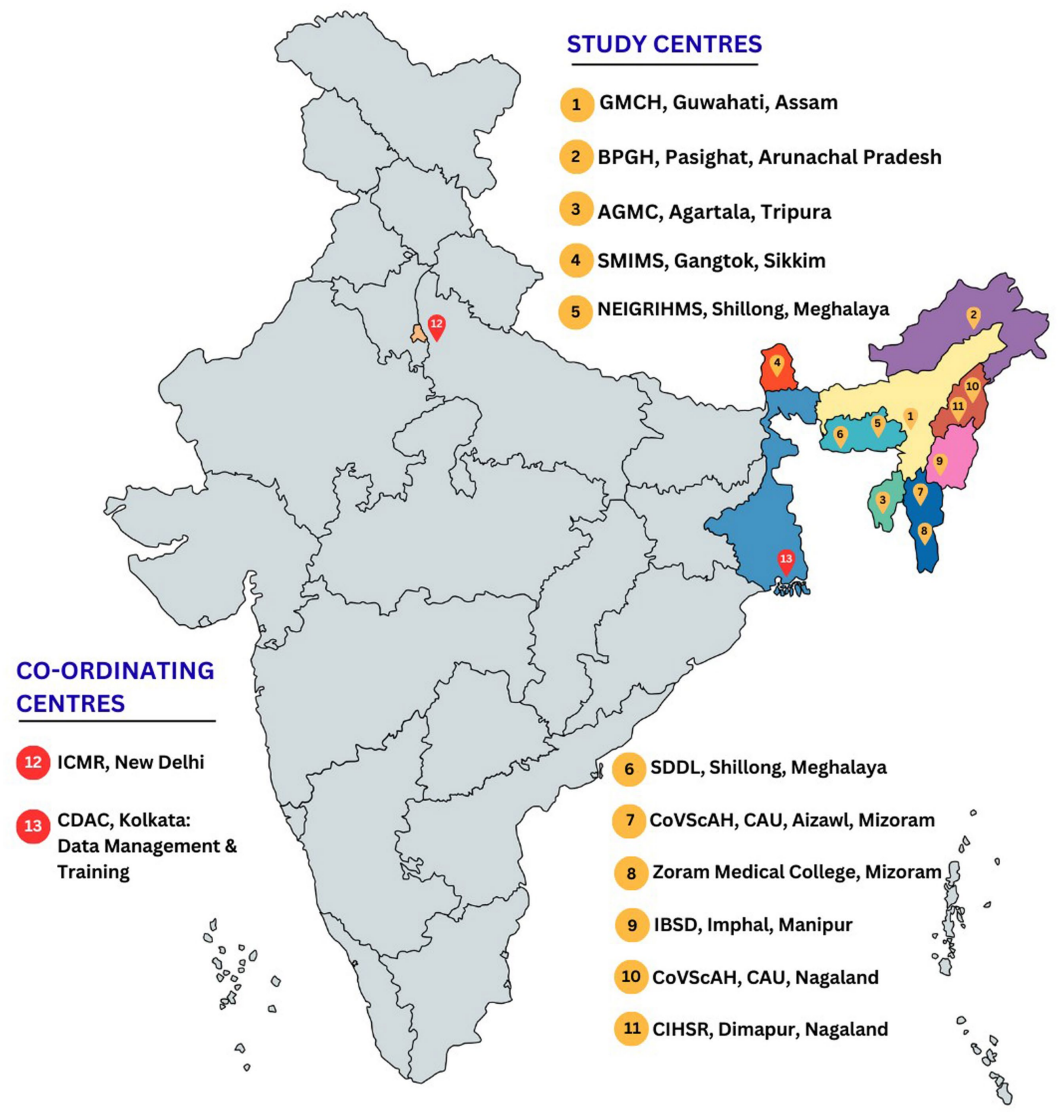
KAP study centres.

The study will cover eight North-Eastern states (Assam, Arunachal Pradesh, Sikkim, Tripura, Manipur, Meghalaya, Mizoram, and Nagaland) of India (Figure 2). Although not randomly assigned, one area in each state will serve as a comparison (non-intervention) group to allow for assessment of intervention impact. The area, from where the intervention (test) groups will be selected will be at least 100 km away from the area of the non-intervention (control) groups to minimize the risk of information spillover from the intervention groups (Table 1).

**Table 1:** State-wise KAP study centres.

State	Centre
Assam	Gauhati Medical College and Hospital (GMCH), Guwahati
Arunachal Pradesh	Bakin Pertin General Hospital & Training Centre (BPGH & TC), Pasighat
Sikkim	Sikkim Manipal Institute of Medical Sciences (SMIMS), Gangtok
Tripura	Agartala Government Medical College (AGMC), Agartala
Manipur	Institute of Bioresources and Sustainable Development (IBSD), Imphal
Meghalaya	North Eastern Indira Gandhi Regional Institute of Health & Medical Sciences (NEIGRIHMS), Shillong
	Meghalaya, State Disease Diagnostic Laboratory (SDDL)
Mizoram	College of Veterinary Science and Animal Husbandry (CVSc & AH), Central Agricultural University (CAU), Aizawl
	Zoram Medical College (ZMC), Falkawn
Nagaland	College of Veterinary Science and Animal Husbandry (CoVSAH), CAU (I), Jalukie, Peren,
	Christian Institute of Health Sciences and Research (CIHSR), Dimapur

For the intervention groups, one school and its surrounding locality will be selected for the study so that the locality-specific inferences are drawn at the end of the project in a state and can be relied upon at least for the locality. The study subjects, *viz.,* schoolchildren, homemakers, street food vendors, and consumers, will be recruited from the same area to obtain a snapshot of the representative food-related KAP information of the area. Community leaders in the vicinity of the school/study area will be identified and involved in the study. A similar approach will be applied to select the non-intervention groups. Spatial strategy for selecting the test and control groups is outlined in detail (Table 2).

**Table 2:** Spatial strategy for control and test study groups.

State	Centre	Test group (T)		Control group (C)		Distance between T & C km (approx.)
		School	Locality	School	Locality	
Assam	GMCH	Dispur Government Higher Secondary School	Dispur	Nagaon Government Boys’ Higher Secondary School	Nagaon	113
Arunachal Pradesh	BPGH&TC	Vivekananda Kendra Vidyalaya, Oyan	Oyan, East Siang	Vivekananda Kendra Vidyalaya, Roing	Lower Dibang Valley, Roing	124
Sikkim	SMIMS	Government Senior Secondary School	Luing	Government Senior Secondary School	Rhenock	103.5
Tripura	AGMC	Mandai Hs School	Mandai	Jalefa Hs School	Jalefa	110
Manipur	IBSD	Awang Sekmai Nongthonband Girls High School,	Awang Leikung Thabi, Sekmai	Holy Child English High School, Kakching	Serou	102
Meghalaya	NEIGRIHMS	Pdeng Raid Border Area Secondary School	East Khasi Hills	Lebiwell Memorial Secondary School	Ri Bhoi	105
	SSDL	Nehru Memorial Higher Secondary School	Umsning	Jirang Government Higher School	Jirang	113
Mizoram	CVSc&AH	Falkawn Middle School, Falkawn,	Falkawn, Aizawl	Baptist English School, Serkawn	Serkawn, Lunglei, Mizoram	145
	ZMC	Kawnpui Higher Secondary School	Kawnpui Hmar Veng	SYNOD Higher Secondary School	Mission Vengthlang	111
Nagaland	CoVSc&AH	Baptist Higher Secondary School	Jalukie B	Dainty Buds School, Jotsoma	Kohima	108
	CIHSR	Great Commission Higher Secondary School	Chumukedima	Clark Memorial Higher Secondary School	Impur	200

### Study population

**Figure 3:**
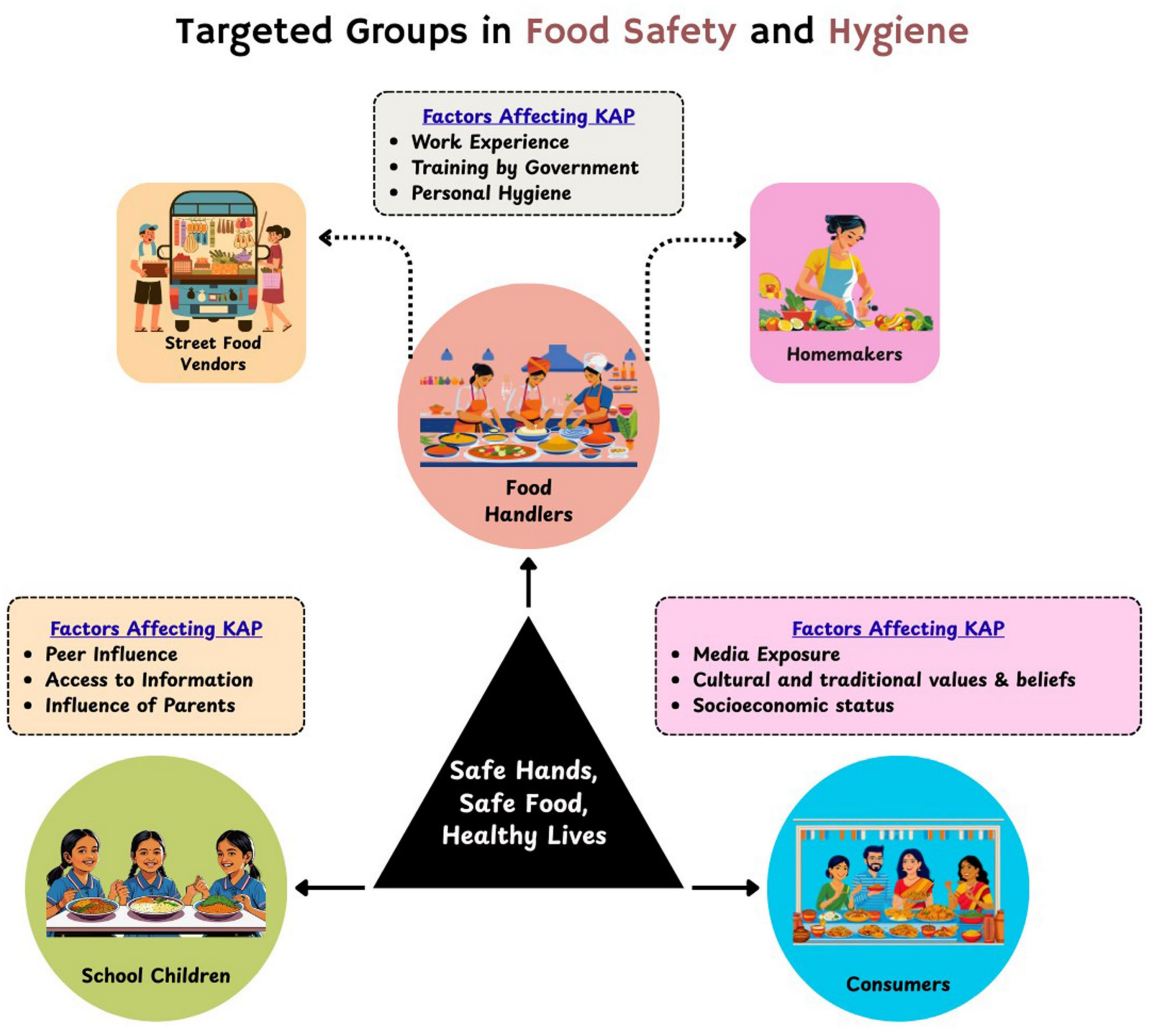
KAP awareness initiative—target groups.

The awareness program is focused on the high-impact groups within the population, i.e., food handlers (homemakers and street food vendors), school children studying in 9^th^ and 10^th^ standard, and consumers (Figure 3). These groups of people play an integral role in overall food safety measures and food safety status of the society. Food handlers, who are directly involved in food processing, preparation, and service, represent a key group with the highest potential to spread foodborne infections due to their central role in the food service. Whereas, school children, the future citizen of the country, are particularly vulnerable to food borne infection, mainly diarrhoeal disease. This underscores the need for them to acquire adequate knowledge about food safety practices. Consumers, likewise, should have awareness about food safety, hygiene, and foodborne infections so that they can identify and refrain themselves from purchasing and consumption of unsafe and unhygienic food items.

### Sample size

A convenient sampling method will be followed for the study. Briefly, in each centre, four (4) different target groups and the respective control groups will be selected. Each target group will consist of 25 study subjects. The control group will consist of at least twelve (12) participants for each corresponding target group, forming an approximate 1:2 ratio with the respective target group. Also, the control group will not be a part of the test target group. There will be an inclusion of 10% buffer for potential drop-outs across all target groups, both test and control. Cooks or attendants of schools involved in mid-day meal programs, wherever possible, will also be included and taken under the Homemaker group. The total sample size for the study is *n* = *1848*, comprising *1,232* test samples and *616* control samples. The pre-post study will be conducted with the same group of *n* = *1848* samples (Table 3).

**Table 3:** Detail sample size for the KAP study.

Sl. No	Sample	Nos.	+10% drop-outs	Total
A	Test target group (T)			
1	School children	25	2.5 ~ 3	28
2	Homemaker & school attendants	25	2.5 ~ 3	28
3	Street food vendors	25	2.5 ~ 3	28
4	Consumers	25	2.5 ~ 3	28
	Total samples/centre (T)	100	12	112
B	Control group			
1	School children	12	1.2 ~ 2	14
2	Homemaker & school attendants	12	1.2 ~ 2	14
3	Street food vendors	12	1.2 ~ 2	14
4	Consumers	12	1.2 ~ 2	14
	Total samples/centre (C)	48	8	56
6	Total study sample size/centre (T + C)	168		
8	Total no. samples in study (from 11 centres)			1848

### Study subject definition

1. *Food handler*: A person who handles packaged or unpackaged food directly as well as the equipment and utensils used to prepare or serve food and/or surfaces that come into contact with food.
2. *Homemaker*: A person who manages the household (such as sewing, cleaning, or cooking) of their own family, especially as a principal occupation.
3. *Cooks/attendants of School*: Personnel involved in the preparation/serving of meals to school students under the mid-day meal program.
4. *Street food vendor*: Vendor that sells and/or prepares ready-to-eat foods and beverages in streets or other public places.
5. *School children*: Children who are enrolled in and attending school in 9^th^ and 10^th^ standard.
6. *Consumers*: Adults who purchase raw or processed food items intended for domestic consumption.
7. *Community leaders*: Adults of social repute who will act as Key communicators in the area of study.
8. *Control group*: Participants, including food handlers, school children, and consumers (as defined above) who will not be exposed to the intervention.

### Inclusion criteria

Apparently healthy people who have willingly provided consent to participate in the study. Food handlers and food service providers including homemakers, personnel of street food vendors, and consumers between the ages of 25–60 years, who have been involved in the respective activities for more than a year will be included. School children between the ages of 12–16 years and attending school in 9^th^ and 10^th^ standard will be included in the study.

### Exclusion criteria

Individuals who are unwilling to participate, those who are reported to be sick or diseased, and adults over 60 years of age will be excluded from the study. Additionally, school children below 12 or above 16 years of age will also be excluded.

### Ethical clearance

Ethical approval has been obtained from Central Ethical Committee on Human Research (CECHR) (Approval letter no. CECHR004/2024), on 19.12.2024. The study protocol, questionnaires, information sheet, consent, and assent forms have also been reviewed and approved by the project review committee and CECHR. Apart from that, each study centre also obtained its institutional ethical clearance. The project was initiated on 1st Feb 2025.

### Informed consent

Written informed consent will be obtained from each participant aged ≥18 years. Data from the School children groups aged 12–16 years (controls and test groups) will be collected after obtaining ‘written consent’ or ‘thumbprint’ from parents in front of class teacher/witness. Verbally recorded consent will be obtained in presence of witness, where written consent is culturally inappropriate or not feasible. Additionally, assent from the School children will also be obtained in a manner that is appropriate for their understanding.

### Confidentiality and compensation details

All information and data obtained from the study will be considered confidential. The digital documentation of the data will be presented in an anonymized fashion. Non-identifiable data (e.g., name and date of birth) will be entered into the database. There will be no pseudonyms, which would make a retrospective re-identification of the study subjects possible. All data and results will be stored for at least 10 years after the publication of the results. Participants will not receive any compensation for their participation in this study.

### Tool for data collection: survey questionnaire

Three sets of questionnaires are tailor-made for each group of participants (consumers, students, street food vendors, and homemakers) to assess the knowledge, attitude, and practice of food safety and hygiene practices based on different studies conducted globally. The questionnaire has been adapted primarily from the WHO (14) guidebook, Centers for Disease control and prevention (CDC) (15), and the WHO’s Five Keys to Safer Food Guidebook (16).

Each e-questionnaire is divided into four sections: Sociodemographic, Knowledge, Attitude and Practices. The sociodemographic section collects participant details such as age, gender, education level, and profession. The knowledge section includes questions covering personal hygiene, cleaning and sanitation, cross-contamination, food preservation, training in cooking, and understanding of foodborne infections. The attitude section evaluates participants’ perceptions and attitudes towards sanitation, hygiene practices, food handling and prevention of foodborne diseases. The practice section assesses participants’ daily practices on food hygiene and safety for prevention of foodborne diseases. Theoretical attitudes can be drawn from respondents’ answers that can portray the big picture of their perspectives. E-questionnaire with a drop-down menu has been developed by CDAC Kolkata, the data management partner of the study.

The questionnaires for the study are carefully designed and there are separate questionnaires for the three different groups. The questionnaire meant for consumers has certain questions tailored to the needs of the consumers (example – questions covering the aspects of expiry dates, importance of buying food from hygienic outlets, etc.). In the same vein, the questionnaire designed for food handlers (street food vendors and homemakers) includes a greater focus on hygiene-related practices, such as maintaining clean workstations and using separate chopping boards for raw and cooked food items. The questionnaire designed for the school children emphasizes on the situations that they face in their daily lives and school environment.

### Field validation of questionnaire

The adapted KAP questionnaires, before field application, have undergone testing for reproducibility to mitigate issues such as recall bias and imprecise answers. Test–retest reliability was evaluated with 10 randomly chosen participants, who were not part of the study sample, with a retest conducted 15 days after the initial administration.

### Intervention tools

Standard Information, Education, and Communication (IEC) materials like WHO’s Five Keys to Safer Food Manual (14), CDC’s Hand Hygiene and Four Steps to Food Safety,^1^ United States Department of Agriculture’s (USDA) “Food Safety: A Need-to-Know for Those At Risk,” FSSAI’s “Keeping Fruit and Vegetables Clean”,^2^ etc. will be tailored to ensure suitability, acceptability, and ease of comprehension for participants. These materials will be adapted to local contexts and disseminated through culturally appropriate channels, including posters, pamphlets, and small group sessions, ensuring they are available in regional languages, *viz.,* Assamese (Assam), Bengali (Tripura), Hindi (Arunachal Pradesh), Khasi (Meghalaya), Meiteilon (Manipur), Mizo (Mizoram), Nagamese (Nagaland), and Nepali and Hindi (Sikkim) for clear comprehension. Interactive sessions will be employed to encourage active participation and adoption of safer hygiene practices.

The IEC materials are aligned with the questionnaire used in the pre-intervention phase, addressing participants’ KAP. They aim not only to introduce new information on food safety and hygiene but also to reinforce and expand participants’ pre-existing knowledge by comparing it with pre-intervention responses.

Participants will receive easy-to-understand fact sheets and tip sheets on essential food safety practices like cleaning, separating, cooking, and chilling. Infographics will visually guide safer food choices, especially when dining out, by highlighting risks. Workshops and interactive lectures will provide practical demonstrations covering handwashing, washing meat, fruits, and vegetables, safe water identification, identification of spoilt food items, proper storage of perishable food, safe handling of raw and cooked foods, and maintaining clean food preparation areas. These sessions aim to enhance knowledge, attitudes, and practices, reduce contamination risks, and promote sustainable hygiene habits. The intervention materials have been thoughtfully designed to accommodate the needs of various target groups and will include visual tools such as cartoons and infographics to facilitate easier comprehension and long-term retention. Certain demonstrations like distinguishing clean water from murky water, proper handwashing technique, etc. will also be carried out to enhance the engagement of the study participants.

### Pre-intervention data

Pre intervention baseline data from the target and control groups will be collected using the standardized questionnaire. Each participant will be assigned a unique alphanumeric ID for tracking purposes. Interviews will be conducted individually, with responses recorded in the app-based electronic questionnaire/Case Report Forms (eCRF) and stored in a centralized database maintained by C-DAC for analysis.

### Intervention

Following the pre-intervention study, food hygiene-related workshops will be conducted for different test groups over 3 weeks, jointly by the research team, IDSP, and the State health team. Intervention tools will be used for the training along with demonstration of food safety measures. The intervention materials are thoughtfully developed to address all aspects covered in the questionnaire. The coordinating centre has organized two online training sessions to equip the field workers for conducting the KAP study at their respective centres. Additionally, training has been provided on implementing the awareness program and recording details of every site visit in an online monitoring portal for continuous assessment of the program.

For the school test groups, a one-two day intervention will be organized where the field workers from different centres will train the children in all aspects of food safety and hygiene. Posters will be put up in the classrooms and pamphlets will also be distributed to ensure dissemination of uniform information across all the 11 centres. Key practices such as proper handwashing techniques, identifying clean water sources, and correctly washing fruits and vegetables will be demonstrated. For the other groups, intervention will be carried out in a similar manner with the help of stakeholders. Community Health Officers and Accredited Social Health Activists (ASHAs) will also be engaged in the program to facilitate door-to-door awareness sessions for homemakers that are unable to attend the main program. The fidelity checks will be done by the co-ordinating team to observe adherence to the protocol, the delivery of the intervention and the quality via individual interactions with the field workers. The centres are also instructed to maintain a checklist containing information about the score of topics covered vs. intended, and photographic records of their KAP study site visits as evidences.

Spatial strategies will be employed to prevent information exchange between the test and control groups.

### Post-Intervention Data

Post-intervention assessments will be conducted for both test and control groups at 1 month (short term) and 6 months (long term) after the intervention. The same eCRF will be used to record and evaluate any temporal changes in knowledge, attitude, and practice concerning food safety. Data collection at these intervals will provide insights into the acceptance, adaptation, and retention of the introduced practices.

### Data management

Data collected and recorded using a centralized app-based system, will be jointly managed by ICMR Hqrs and C-DAC Kolkata. Each participating centre will have secure access through unique login credentials assigned specifically for their use.

The data management system is equipped with stringent quality control measures, including automated checks to identify and flag duplicates, missing data, and inconsistencies. Data entry will follow predefined protocols to maintain uniformity and reduce errors. Furthermore, the platform supports basic descriptive analytics and report generation, facilitating preliminary insights at the local level.

Data completeness and accuracy will be routinely monitored through regular reviews, and discrepancies will be resolved in consultation with the respective centres. To protect confidentiality and adhere to ethical standards, all data will be safely kept in a centralized database with the proper encryption and access control mechanisms in place. This structured approach ensures reliable data collection and supports rigorous analysis for meaningful outcomes. Both online and offline training for data entry will be provided to all site investigators and technical personnel.

### KAP questionnaire: organization and scoring

The KAP assessment is divided into three blocks, *viz,* (i) Knowledge Block: Contains 30 multiple-choice questions on personal hygiene, food hygiene, cross-contamination, and food thawing. Correct answers will be awarded one point, while incorrect, or “I do not know” responses receive zero points. The score range for this section is 0 to 30. (ii) Attitude Block: Comprises 20 questions of the Agree/disagree type, assessing the importance of hygiene, food handlers’ responsibility, and ongoing food safety training. Attitudes are considered indicators of behaviors, and responses are scored on a scale from 0 to 20 points and, (iii) Practice Block: consists of 15 questions to evaluate how participants adopt and practice food safety and hygiene practices in their everyday lives. The scoring in this section ranges from 0 to 60 points corresponding to the ordinal responses and is based on a Likert-type scale. The total scores of all the sections will be then converted into percentages and categorized based on Bloom’s cut-off points (Good: 80–100%, Moderate: 60–79%, Poor: <60%). The participants will require approximately 15 min to complete the questionnaire, and will be assisted by investigators who will read questions aloud without offering additional explanations. In case of uncertainty, questions may be repeated, or participants can read them independently. An overall score of 70% or higher will be considered satisfactory, based on criteria by Soares et al. (17).

### Data analysis and interpretation

Data will be captured using Computer-Assisted Personal Interviewing (CAPI). During interviews, this digital method allows direct entry of field data into electronic devices such as tablets or smartphones. The collected data will then be systematically transferred and formatted for statistical analysis, data management, and visualization. Popular statistical software tools such as IBM Statistical Package for the Social Sciences (SPSS) (Version 31) will be used for the analysis of the data of the KAP survey.

Comparative analysis between the control and test groups will be done by using appropriate statistical methods. The scores of the Knowledge, practice and attitudes will be presented as frequencies and percentages for questions having the categorical yes/no options. The continuous variables will be presented as means and standard deviations.

For the analysis of the pre- and post-intervention scores in case of categorical variables, Mc Nemar’s test will be used. Chi-square test will be used for the comparison of proportions across different groups (example—for comparison of scores of the street food vendors, test vs. controls). The *p*-value <0.05 will be considered as statistically significant. For comparing continuous variables between different groups with normally distributed data, an independent samples *t*-test will be applied. In cases where the data is not normally distributed, the Mann–Whitney U test will be employed. For the comparison between continuous variables of the pre- and post-intervention data, a paired t-test (for normally distributed data)/Wilcoxon signed-rank (for skewed data) shall be used.

The effectiveness of the intervention will be determined from the post-intervention data. This will ensure that the observed improvements in KAP scores among test participants resulted from the intervention itself.

### Data use and dissemination of findings

A report of the survey findings will be communicated to the FSSAI, and Ministry of Health and Family welfare (MoH & FW) for the necessary policy guidance for the country. Study results and important findings will be disseminated to all relevant stakeholders.

### Study progress

The study was initiated on 1st February 2025 and implemented in all 11 centres. Several critical milestones have been achieved, reflecting significant progress towards the study’s objective of enhancing food safety and hygiene awareness across the 8 North-Eastern states of India. A key initial step involved the development of comprehensive sets of questionnaires to capture essential aspects of KAP related to food safety and hygiene. These questionnaires have been tailored to suit the diverse target groups, ensuring their relevance and effectiveness.

A well-structured Participant Information Sheet has been developed, providing detailed explanations of the study’s objectives, methods, and ethical considerations, to complement the data collection tools. The information sheet emphasizes informed consent and voluntary participation, ensuring transparency, absence of risk, and freedom to withdraw without any penalty. Accompanying this, informed consent, parental/LAR assent and written consent forms, have been developed in accordance to ethical research standards.

An important milestone in the study was securing all necessary approvals from the Technical Review Group (TRG), the Competent Authority at the Indian Council of Medical Research (ICMR), and ICMR Central Ethics Committee on Human Research (CECHR). Such sanctions highlight the study’s compliance with strict scientific and ethical guidelines, further solidifying its credibility. All training materials, and consent forms, were translated into eight major local languages to suit the linguistic and cultural diversity of the North-Eastern states. This initiative ensured accessibility and effective communication with participants, fostering inclusivity and engagement across the targeted populations.

To enhance field data collection efficiency, the questionnaires were adapted into electronic format (eKAP) compatible with Android-based smart devices. This innovative eKAP format facilitates a streamlined data management through robust, reliable real-time data recording and synchronization to a central database.

The study has also raised community awareness through effective food safety awareness programs conducted in Jalukie and Dimapur districts of Nagaland, and Shillong in Meghalaya. These outreach programs involved community members as well as school children, to foster increased understanding and the adoption of safe food handling practices. Such initiatives have already started to create positive impact within these communities.

Another noteworthy accomplishment was the pre-testing of the eKAP questionnaires, conducted in two separate states and different target groups. The first version of the mobile-based e-questionnaire (eKAP) was pre-tested among 30 participants, comprising 10 school children in Peren, Nagaland, and 10 street food vendors and 10 consumers in Shillong, Meghalaya. During this phase, positive engagement was observed, and several practical insights were gained. In the school children group, improvements in clarity of questions and interface readability were identified and incorporated as the field workers reported that few scientific terms used in the questions were challenging for the children. Among vendors and consumers, the digital tool was found to be user-friendly, with minimal assistance required. Participant feedback led to modifications and reformulation of the questionnaire. The average time required to complete the form was approximately 15 min. The app’s built-in validation features were effective in preventing missing or illogical entries. These findings confirmed the feasibility, clarity, and practical usability of the tool (Mobile App) across the intended study groups.

Another notable accomplishment of the study was fostering collaboration and active networking with state health agencies and regulatory bodies, including the IDSP, State Health Officers (SHO), FSSAI, and local food safety officers. Engaging these stakeholders has been pivotal for effective implementation of the study and awareness programs. Their involvement not only enhanced the credibility and reach of the initiatives but also ensured alignment with pre-existing regulatory frameworks and public health strategies. This collaborative approach emphasizes the importance of leveraging institutional support for sustained impact.

These accomplishments collectively highlight the study’s commitment to a meticulous and community-centred approach. By integrating tailored tools, inclusive practices, and innovative technologies, the study has established a robust foundation for the subsequent phases of its research endeavors. The study result is expected by January 2026.

## Discussion

The North-Eastern states of India are well-known for their distinctive geographical, historical, cultural, and traditional diversity as well as their distinctive culinary practices. The region is home to over 200 distinct ethnic groups, all of whom have followed traditional food habits, with fermented foods, bamboo shoots, edible insects, and smoked meat forming a central part of their diets (18). Although these foods contribute to cultural identity and nutrition, their traditional preparation methods often lack established food safety practices, posing potential health concerns (19). Although food and water borne diseases and outbreaks are recognized as a public health problem in NE India, data from NE India on food and water borne diseases is limited. A surveillance conducted during 2020–23, reported an overall sample positivity of 3.1% for food borne pathogens in different food items. Also, 8.9% enteric pathogens positivity was reported in the rectal swab samples of hospitalised diarrhoeal cases (19).

Studies have shown that incorrect handling and microbial contamination during process of preparation like fermentation, smoking, drying, etc., can lead to the growth of pathogens like *Salmonella, Listeria,* and *Clostridium botulinum* (20, 21). Such concerns are compounded by inadequate knowledge and awareness of food hygiene practices in the region, highlighting the need for targeted interventions.

Foodborne illnesses and the rising menace of AMR are major public health concerns on a global scale. Millions of people are impacted each year, resulting in significant illness, mortality, and financial losses across the world (22). As per WHO report, in Africa, nearly 92 million people fall sick due to the consumption of contaminated foods, resulting in 1,37,000 fatalities each year (23). Contaminated food is responsible for about 70% of diarrhoeal diseases in Ethiopia, while food handlers account for 10–20% of food-borne disease outbreaks through contamination (24, 25). In India, the challenges are made even tougher by poor food safety practices, inadequate sanitation, and the rising issue of AMR, especially in remote and under-resourced areas like the North-eastern states. Tackling these problems calls for a comprehensive strategy, where KAP studies are crucial in pinpointing gaps in understanding the behaviors towards food hygiene and safety. For instance, a recent study in a rural part of Tamil Nadu, India found that food handlers with poor handwashing habits and kitchens infested with pests faced a higher risk of diarrhoeal diseases (26). Similarly, a cross-sectional study of food handlers in semi-urban Lucknow, India showed that only half of the participants wore gloves while preparing and handling food (27). Additionally, findings from a 2021 study in four villages in Bangladesh revealed that most women involved were unaware of the dangers of leaving cooked meals at room temperature for too long (28). In Karnataka, a KAP study referencing the WHO’s “Five keys for food safety” guidelines indicated that while many participants knew about good handwashing practices, only 44% understood the importance of thoroughly reheating cooked food (29). Such evidence-based findings, highlight the urgent need for implementing awareness campaigns and structured interventional programs aimed at improving sanitary hygiene and food safety practices within communities.

Awareness as well as informed practices regarding food safety and hygiene are crucial for preventing and controlling foodborne illnesses, and the alarming AMR menace. KAP studies have provided valuable insights into behavioral and cultural factors that influence food hygiene practices, consequently facilitating development of targeted interventions. Studies from Nigeria showed that community-based KAP programs positively influenced food hygienic practices, and effectively reduced incidences of diarrhoea among participants (30). Interventions following culturally tailored educational campaigns in rural China showed increased adoption of safe food handling techniques. Another study in Brazil also showed significant reduction in bacterial contamination in community kitchens after targeted food safety and hygiene trainings (31). These findings indicated the crucial role of integrating behavioral studies into public health strategies.

Evidence from previous research highlights just how impactful KAP studies can be in public health. For instance, pre-post-intervention studies conducted in Bangladesh and Kenya demonstrated significant improvements in food safety practices, resulting in reduced incidences of foodborne diseases (32). These findings validate the effectiveness of combining educational interventions with scientific assessments. The current program brings these elements together, ensuring that participants are well-informed while also addressing the systemic issues in food hygiene practices. Over time, the program anticipates to see measurable decreases in foodborne disease prevalence and AMR, leading to better public health outcomes.

The ICMR-FoodNet program has played a crucial role in generating important epidemiological data on foodborne pathogens and AMR pattern in India. Its surveillance-based approach, particularly in the North-eastern states, has filled important gaps in pathogen monitoring and profiling (18, 33, 34). While the contributions of ICMR-FoodNet are indispensable, it is also essential to focus on the human aspect of food safety—like knowledge, attitudes, and practices—as this adds a vital layer to our public health response. To date, very few KAP studies related to food safety have been reported in the North-Eastern states and are mostly restricted to the states of Assam and Sikkim.

A study conducted in Guwahati, Assam, revealed that only 30–37% of street food vendors followed safe food handling practices (35). However, a subsequent study conducted by the same team in 2011 showed a notable shift in the knowledge and practices of street food vendors after they underwent food safety and hygiene training sessions. The knowledge level of the food vendors increased dramatically from an average of 24.35–66.2%, and their practice scores improved by 37.5–50.8% after the intervention (36). In another questionnaire-based study among 100 random street food vendors in semi-urban and urban areas of Guwahati, Assam, researchers uncovered several concerning practices. For instance, the vendors were found to have unclean nails, used unsafe water for cooking, and resorted to improper storage of left-over food (37).

A recent pre-post KAP study conducted in Gangtok, Sikkim used a self-instructional module as the intervention strategy to raise awareness about safe food handling. The findings demonstrated a significant improvement in the food hygiene knowledge of the fast-food handlers (38). The present KAP awareness initiative complements ICMR-FoodNet by addressing behavioral and cultural determinants linked to foodborne illnesses and creating a holistic strategy that blends scientific data with community-centred interventions. This represents a crucial step, towards sustainable public health advancements. Data collection at three different time points will allow the researchers to track changes, trends, or developments in knowledge, attitudes, and practices of the different target groups after the intervention.

The WHO’s *Global Strategy for Food Safety 2022–2030* emphasizes the importance of integrating education and behavioral change into broader surveillance and intervention frameworks (39), highlighting the demand for coordinated international efforts to mitigate the global challenges of foodborne illnesses and associated AMR. The present KAP initiative aligns with this global vision, prioritizing culturally sensitive interventions that address local realities while also contributing to international food safety goals.

The lessons learned from this initiative in Northeast India could be instrumental in shaping similar initiatives in other low and middle-income countries, showcasing its global relevance. The findings from this comprehensive KAP study underline the critical role of targeted interventions in tackling food hygiene challenges in the North Eastern states of India. The pre-post intervention strategy demonstrates the significant potential for improving food safety awareness and practices among diverse groups of the community. Fostering better community engagement and stakeholder collaboration can effectively facilitate the successful dissemination and adoption of safe food practices. Moreover, the study will shed light on the traditional food preparation methods and the risks they carry, stressing the importance of culturally sensitive educational programs. By aligning with the ongoing initiative of ICMR FoodNet program, the study sets the stage for reducing the burden of foodborne illnesses through informed policy-making and community-driven solutions. Future efforts should focus on scaling up these interventions throughout India to ensure widespread improvements in public health.

### Expected outcome

The mobile app-based Electronics Case Report Form (eCRF) will help in capturing real time data. This system has been designed to reduce data entry errors and facilitate automated filtering of flawed or incomplete responses. The field investigators have been trained extensively to ensure uniform use of the app across all centres. The eCRF is synchronized to a centralized database, allowing quality control to be maintained through automated checks for inconsistencies, duplications, and missing values. All these digital mechanisms will facilitate in exploring the sparsely researched food habits and practices in the North Eastern states of India. On a larger scale, the awareness program may also help to enhance food safety in the study regions and promote better health. This initiative has the potential to serve as a scalable model for similar initiatives both nationally and internationally, aligning with public health goals and contributing to the global fight against foodborne diseases. Findings from the study have the potential to shape the future food safety policy and foodborne illness prevention programs.

### Future prospect

The valuable insights gathered from the study can be instrumental in crafting a strategic Social and Behavior Change Communication (SBCC) model tailored for mass education, awareness, and the promotion of food safety and hygiene practices. This data-driven approach can serve as a robust evidence-based system for state governments and healthcare stakeholders to develop policies and programs that effectively tackle region-specific gaps in knowledge, attitudes, and practices pertaining to food safety and hygiene.

### Limitation

The study is geographically restricted to the North-East region of India, because of their unique food habit. Additionally, children below 12 years of age were excluded, as they might be unable to understand and retain intervention information, which may leave out valuable insights into the KAP of this demographic, particularly concerning food safety and hygiene education. The study primarily relies on self-reported data, which may introduce certain biases like social desirability bias, where participants might respond in a manner that they believe is socially acceptable rather than reflecting their true beliefs. Despite receiving training, field workers may unintentionally influence responses through the way in which questions are delivered.

The study’s focus on immediate post-intervention outcomes may also limit insights into long-term behavioral changes (beyond 6 months). Capturing such changes would require regular follow-ups, which could be undertaken by IDSP and State Health teams. Another limitation of the study may be that due to the community-based nature of the program, there is a possibility of cross-exposure, where control group participants may be indirectly exposed to interventions originally intended solely for the test group.

## Footnotes

^1^ https://www.cdc.gov/clean-hands/about/index.html

^2^ https://fssai.gov.in/
